# Cardiac Structure Relates to Hemorrhagic Cerebral Small Vessel Disease Phenotype

**DOI:** 10.1161/JAHA.124.039474

**Published:** 2026-02-11

**Authors:** Catriona R. Stewart, James Lyon, Philip S. Nash, Jonathan G. Best, Guendalina Bonifacio, Jukrapope Jitpimolmard, Rhys P.D. Inward, Edgar Chan, Rupert Oliver, David J. Werring

**Affiliations:** ^1^ Department of Translational Neuroscience and Stroke, Stroke Research Centre, UCL Queen Square Institute of Neurology University College London, and National Hospital for Neurology and Neurosurgery, University College London Hospitals NHS Foundation Trust London UK; ^2^ Department of Neurology University Hospital Southampton Southampton UK; ^3^ Department of Neurology Ratchaphruek Hospital, Mueang Khon Kaen Thailand; ^4^ Department of Biology University of Oxford UK; ^5^ Pandemic Sciences Institute University of Oxford UK

**Keywords:** arteriolosclerosis, cerebral amyloid angiopathy, cerebral small vessel disease, intracerebral hemorrhage, left ventricular mass, neuroimaging, Intracranial Hemorrhage

## Abstract

**Background:**

Most intracerebral hemorrhages (ICH) are caused by 1 of 2 cerebral small vessel diseases (cSVDs): arteriolosclerosis and cerebral amyloid angiopathy (CAA). Hypertension is a major risk factor for ICH, but its contribution to the hemorrhagic manifestations of these arteriopathies remains uncertain. We investigated associations between a cardiac structural biomarker of systemic hypertension (left ventricular mass [LVM]) and cSVD neuroimaging phenotype in patients with ICH.

**Methods:**

We assessed brain magnetic resonance imaging and echocardiography cross‐sectional data from patients with symptomatic hemorrhagic cSVD, including macroscopic ICH, convexity subarachnoid hemorrhage, or cognitive impairment. We compared LVM in patients with possible or probable CAA, mixed pattern cSVD, or arteriolosclerosis. We used linear regression models to investigate associations between LVM, patient characteristics, and SVD.

**Results:**

We included 216 patients (104 with CAA, 91 with mixed pattern cSVD, and 21 with arteriolosclerosis). Patients with CAA had a significantly lower mean LVM (148.8±44.9 g) compared with those with mixed pattern cSVD or arteriolosclerosis (172.8±59.3 g) (*P*<0.001). Across all SVD classifications, LVM progressively increased: CAA (148.8±44.9 g), mixed pattern cSVD (168.7±55.3 g), and arteriolosclerosis (190.8±72.9 g). In a multivariable linear regression model adjusted for age, sex, and hypertension, LVM was independently associated with CAA (adjusted mean difference in LVM, 14.6 [95% CI, 1.7–27.4] g higher for mixed pattern cSVD or arteriolosclerosis compared with CAA, *P*=0.026).

**Conclusions:**

Our findings suggest cardiac structure relates to the neuroimaging phenotype of symptomatic hemorrhagic cSVD, including ICH. This is relevant to the classification, understanding, and prevention of hemorrhagic cSVD.

**Nonstandard Abbreviations and Acronyms**
CAA: cerebral amyloid angiopathy
cSVD: cerebral small vessel disease
ICH: intracerebral hemorrhage
LVM: left ventricular mass

**Clinical Perspective**

**What Is New?**

- In a population with symptomatic cerebral small vessel disease (cSVD), left ventricular mass was independently associated with cerebral small vessel arteriopathy phenotype, with a higher mass predicting mixed pattern cSVD or arteriolosclerosis and a lower mass predicting cerebral amyloid angiopathy as the underlying pathophysiology.

**What Are the Clinical Implications?**

- In patients with cSVD, lower left ventricular mass suggests that further investigations should be done to detect cerebral amyloid angiopathy.
- If patients have symptomatic mixed cSVD, higher left ventricular mass suggests that the underlying small vessel arteriopathy is more likely to be arteriolosclerosis and that vascular risk factor control is of the utmost importance to prevent stroke and progression of cSVD.

Diseases of the cerebral small vessels are the most frequent underlying cause of spontaneous intracerebral hemorrhage (ICH).^1^ The main types of sporadic small vessel disease (SVD) are arteriolosclerosis (previously termed deep perforator arteriopathy, which affects deep perforating arterioles supplying the basal ganglia, deep hemispheric white matter, and brainstem), and cerebral amyloid angiopathy (CAA, which affects mainly superficial leptomeningeal and cortical arterioles), which, together, account for approximately 77% of ICH.^2^

Classification of the underlying cause of ICH is important because CAA‐associated ICH carries a higher risk of recurrent ICH and poststroke dementia compared with ICH with underlying arteriolosclerosis.^3^ Magnetic resonance imaging (MRI) features associated with CAA include lobar cerebral microbleeds, enlarged perivascular spaces in the centrum semiovale, convexity subarachnoid hemorrhage, and cortical superficial siderosis,^4^ whereas deep cerebral microbleeds, lacunes of presumed vascular origin, and basal ganglia enlarged perivascular spaces are associated with arteriolosclerosis. Thus, using brain imaging biomarkers, patients with ICH can be classed as having underlying probable or possible CAA or arteriolosclerosis, although recent postmortem evidence suggests that many patients with ICH have both CAA and arteriolosclerosis pathologies.^5^

Although hypertension is the strongest modifiable risk factor for ICH, the anatomical and pathological differences between arteriolosclerosis and CAA might mean that they are differentially affected by systemic hypertension, with potential implications for understanding ICH mechanisms and prevention. A progressive increase in the stiffness of large arteries, often associated with aging and hypertension, can subject the deep small vessels of the brain to higher pulse pressure, which can subsequently encourage arteriolosclerosis and progressive white matter injury.^6^ Various studies have shown an association between hypertension and arterial stiffness with cerebral SVD (cSVD) features, including lacunes^7^ and enlarged perivascular spaces in the basal ganglia.^7^, ^8^

By contrast, CAA is mainly due to amyloid‐beta deposition in superficial small vessel walls, eventually replacing smooth muscle cells, causing vascular fragility and dysfunction that are hypothesized to lead to intracranial hemorrhage.^9^ Impaired perivascular drainage contributes to the buildup of amyloid‐beta in the perivascular space and subsequent deposition within small leptomeningeal and cortical arteries.^9^ The hallmark of leptomeningeal CAA, cortical superficial siderosis, is hypothesized to be due to previous acute convexity subarachnoid hemorrhage^10^, ^11^; as acute blood products are degraded over time, blood residues are left in the subpial layers of the superficial cortical cerebral convexities.^12^ Because CAA is mainly a disease of impaired clearance of amyloid‐beta, it might not be expected to be influenced to such a large extent as deep perforator arteriopathy by systemic hypertension.^13^

Cardiac structural changes may provide useful quantitative biomarkers of previous exposure to systemic hypertension with relevance for understanding how hypertension might influence the pattern of cSVD. The left ventricle remodels throughout life in response to nonmodifiable and modifiable cardiovascular risk factors. Systemic hypertension is the most important factor involved in remodeling by inducing myocyte hypertrophy and interstitial fibrosis, leading to left ventricular hypertrophy (LVH), which can be detected by echocardiography. LVH, considered a marker of the burden (ie, severity and duration) of systemic hypertension, is characterized by an abnormal enlargement of the left ventricular mass (LVM).^14^ LVM, a continuous variable, can identify more subtle changes, whereas LVH is a commonly used simple clinical measure of pathologically high LVM.

## Aims and Hypotheses

We investigated cardiac structure using echocardiography and the spectrum of cSVD using brain MRI, in a cohort of patients with symptomatic hemorrhagic cSVD seen in a specialist clinic. We hypothesized that LV structural abnormalities (ie, higher LVM) are less severe in patients with possible or probable CAA compared with those with mixed pattern cSVD or arteriolosclerosis.

## METHODS

### Data Access Statement

We will consider reasonable requests for anonymized data, but no data can be released without approval by the University College London Hospitals National Health Service Foundation Trust Governance Review Board.

### Study Design and Participants

This is a retrospective, cross‐sectional study of consecutive patients attending the specialist intracranial hemorrhage clinic, between 2016 and 2023, at the National Hospital for Neurology and Neurosurgery, Queen Square, a tertiary center accepting referrals from across the United Kingdom. Patients were eligible for inclusion if they had symptomatic cSVD (including intracranial [ie, convexity subarachnoid hemorrhage or intracerebral] hemorrhage), or cognitive decline attributed to cSVD with hemorrhagic features (eg, cerebral microbleeds); available MRI scans; and quantitative standardized echocardiography reports to calculate LVM. MRI scans and echocardiography were performed on the day of clinic assessment as part of routine care. Patients with ICH with an underlying macrovascular cause (including aneurysm, cavernoma, and arteriovenous malformation) or cryptogenic ICH were excluded. There were no other exclusion criteria. The final sample size was determined according to the number of patients meeting the eligibility criteria.

### Procedures

All patients underwent a brain MRI. The MRI protocol included axial T1‐weighted, axial T2‐ weighted, axial susceptibility‐weighted imaging sequences and coronal fluid‐attenuated inversion recovery (FLAIR) imaging. The analysis of cSVD features on MRI was carried out using validated criteria, scales, and scores, including the Fazekas scale for white matter hyperintensities (within periventricular and deep white matter)^15^; the Microbleed Anatomical Rating Scale for cerebral microbleeds,^16^ a validated 4‐point scale for perivascular spaces (within the basal ganglia and centrum semiovale)^17^, ^18^; the Standards for Reporting Vascular Changes on Neuroimaging definition for lacunes^19^; and the classification of cortical superficial siderosis as focal (≤3 sulci) or disseminated (≥4 sulci) linear residues of chronic blood deposition in the superficial cerebral cortex.^12^ Acute convexity subarachnoid hemorrhage was defined as a linear hypointensity in the subarachnoid space, involving at least 1 of the cortical sulci on susceptibility‐weighted MRI.^12^

Krippendorf’s Alpha for ordinal interrater agreement was 0.88 for cerebral microbleeds, indicating excellent agreement between raters; 0.68 and 0.66 for deep and periventricular white matter hyperintensities, respectively; and 0.68 for perivascular spaces.

**Figure 1:**
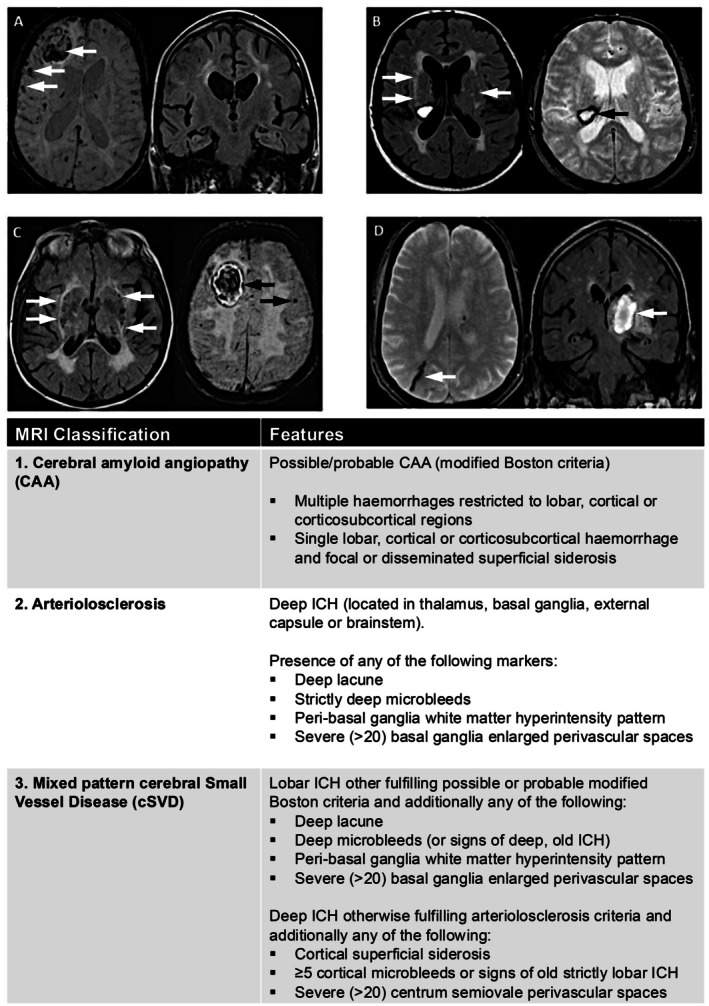
MRI‐based classification of ICH. Images showing (**A**) probable CAA: lobar ICH with lobar CMB (both arrowed), no deep pathology; (**B**) arteriolosclerosis: no lobar CMB/cSS, deep ICH (arrowed) and peri‐BG WMH (arrowed); (**C**) mixed pattern SVD: lobar ICH and lobar CMB, deep and peri‐BG WMH (arrowed) and BG‐PVS; (**D**) mixed pattern SVD: focal cSS (arrowed), deep ICH located in the BG (arrowed). BG indicates basal ganglia; BG‐PVS; basal ganglia perivascular space; CAA, cerebral amyloid angiopathy; CMB, cerebral microbleed; CS‐PVS, centrum semiovale perivascular space; cSS, cortical superficial siderosis; ICH, intracerebral hemorrhage; MRI, magnetic resonance imaging; SVD, cerebral small vessel disease; and WMH, white matter hyperintensity.

Patients meeting the inclusion criteria were classified as CAA, mixed pattern cSVD, or arteriolosclerosis according to an MRI‐based classification of cSVD (Figure 1). For some comparisons, we further simplified this classification to CAA and non‐CAA (ie, mixed pattern cSVD or arteriolosclerosis). CAA was defined according to the modified Boston criteria v2.0 for possible and probable CAA.^20^ Patients with deep ICH (in the thalamus, basal ganglia, external capsule, or brainstem) with deep cSVD features, including deep lacune(s), deep microbleed(s), or severe enlarged perivascular spaces in the basal ganglia (basal ganglia perivascular space rating ≥3 according to the 4‐point scale)^18^ were classified as arteriolosclerosis. Patients with lobar ICH otherwise fulfilling the modified Boston criteria but with any deep cerebral microbleeds, or patients with deep ICH otherwise fulfilling the arteriolosclerosis criteria with CAA‐related cSVD features, were classified as mixed pattern cSVD, as were patients with deep ICH with ≥5 lobar cerebral microbleeds.

LVM was calculated from formal echocardiography reports, in either M Mode or 2‐dimensional tracing, using the following formula proposed by Devereux and colleagues and the American Society of Echocardiography.^21^

$$\begin{array}{c} \mathrm{LV}\;\text{mass}\\ =0.8\;\left(1.04\;\left({\left[\text{LVIDD}+\text{PWTD}+\text{IVSTD}\right]}^{3}-{\left[\text{LVIDD}\right]}^{3}\right)\right)+0.6\;g \end{array}$$

where LVIDD=LV internal diameter in diastole (cm), PWTD=posterior wall thickness in diastole (cm), and IVSTD=interventricular septum thickness in diastole (cm).

Baseline clinical data were collected using a standardized data collection proforma based on diagnoses identified from all information available within the patient’s electronic patient record. Hypertension was defined as either a previous diagnosis or preexisting use of antihypertensive medication. Diabetes was defined as ongoing or newly initiated therapy with antidiabetic drugs or a hemoglobin A1c of ≥6.5%. Dyslipidemia was defined as a known history of abnormal lipid profile, use of lipid‐lowering medication or total cholesterol >5.0 mmol/L on laboratory testing. We did not use high‐density lipoprotein or low‐density lipoprotein cholesterol values to define dyslipidemia. Coronary artery disease was defined as a previous history of myocardial infarction, or stable or unstable angina. Atrial fibrillation was defined as a known history or new diagnosis on ECG. Peripheral arterial disease was defined as a history of limb claudication or intervention for peripheral arterial atherosclerosis. Lifestyle risk factors including previous smoking or excess alcohol consumption (>14 units per week, regularly) were also recorded.

The study was approved by the University College London Hospitals National Health Service Foundation Trust Governance Review Board as a Service Evaluation (registration reference 07‐202324‐SE); because data were collected as part of routine clinical care, the requirement for individual patient consent was waived.

### Statistical Analysis

Numerical data were described using mean±SD and median (interquartile range) depending on variable distributions. Categorical variables were described using number (%). We compared demographic and clinical characteristics as well as neuroimaging features of cSVD between patients in the CAA, mixed pattern cSVD, and arteriolosclerosis groups using 1‐way ANOVA or Kruskal–Wallis tests for numerical variables depending on their distributions and the Fisher’s exact test or χ^2^ test for categorical variables.

We used independent samples *t* tests to compare LVM in patients with CAA and patients with mixed pattern cSVD or arteriolosclerosis, and between patients with CAA with and without macroscopic ICH. One‐way ANOVA and post hoc Tukey tests were used to compare LVM between multiple subgroups (ie, CAA, mixed pattern cSVD, and arteriolosclerosis).

We used linear regression to investigate the relationship between patient characteristics, cSVD, and LVM. Using univariable analysis, we computed beta‐coefficients (β), 95% CIs, and the associated *P* value for potential confounding variables plausibly associated with cSVD and LVM: age, sex, hypertension, diabetes, dyslipidemia, coronary or peripheral arterial disease, current or previous alcohol excess, and current or previous smoking. We then developed a multivariable linear regression model including all variables with a univariable *P* value <0.1. For the 3‐group cSVD classification we used postestimation Wald tests to determine an overall *P* value for the trend. We did not include individual cSVD markers in the models owing to potential collinearity with cSVD classification.

We performed additional ordinal and binary logistic regression analyses with cSVD classification as the dependent variable and LVM and the covariates as the independent variables. We assessed the proportional odds assumption using the Brant test.

Missing data were handled through listwise deletion, given that there was a low proportion of missing data, and our sample size remained robust for analyses. A *P* value ≤0.05 was considered statistically significant.

All statistical analyses were performed by J.L. and P.S.N. using STATA performed in Stata 18 (version 4.4.1).

## RESULTS

### Study Population

**Figure 2:**
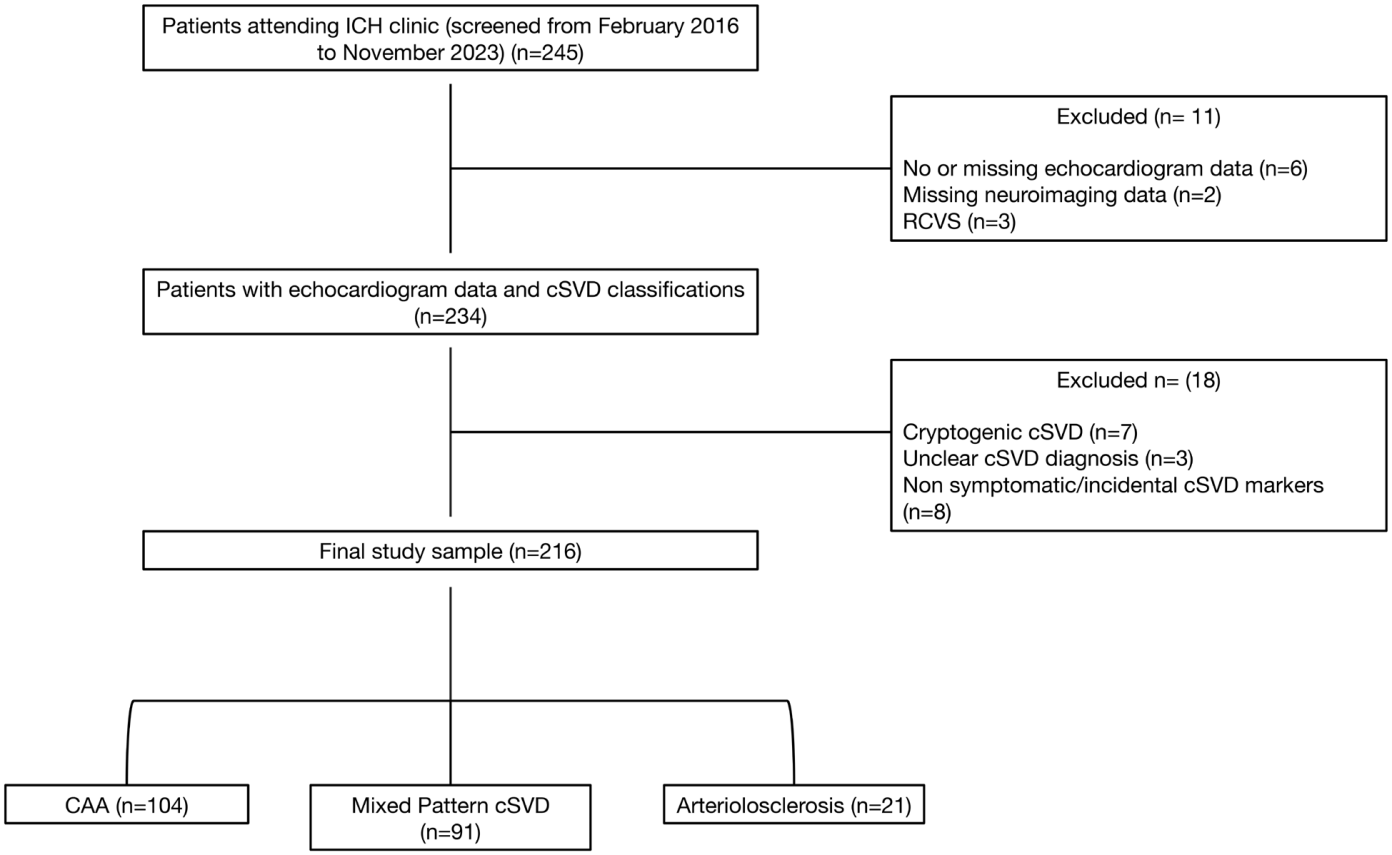
Flow chart of included participants. CAA indicates cerebral amyloid angiopathy; ICH, intracerebral hemorrhage; RCVS, reversible cerebral vasoconstriction syndrome; and cSVD, cerebral small vessel disease.

We included 216 patients. A flow chart showing the included and excluded participants is provided in Figure 2. Participants had a median age of 72 years (interquartile range, 64–77), and 80 (37%) were female. Among these participants 59.7% had hypertension and 8.8% had diabetes (Table 1); 104 participants were classified as CAA, 91 (81.3%) as mixed pattern cSVD, and 21 (18.8%) as arteriolosclerosis.

**Table 1:** Patient Characteristics According to cSVD Classification

	All (n=216)	CAA (n=104)	Mixed cSVD (n=91)	Arteriolosclerosis (n=21)	*P* value
Age, y, median (interquartile range)	72.0 (64.0–77.0)	72.5 (67.5–77.0)	71.0 (63.0–77.0)	56.0 (43.0–65.0)	<0.001
Sex, female	80 (37.0%)	39 (37.5%)	37 (40.7%)	4 (19.0%)	0.179
Hypertension	129 (59.7%)	46 (44.2%)	64 (70.3%)	19 (90.5%)	<0.001
Diabetes	19 (8.8%)	10 (9.6%)	7 (7.7%)	2 (9.5%)	0.887
Dyslipidemia	61 (28.2%)	31 (29.8%)	24 (26.4%)	6 (28.6%)	0.868
Coronary or peripheral arterial disease	49 (22.7%)	21 (20.2%)	25 (27.5%)	3 (14.3%)	0.301
Atrial fibrillation	25 (11.6%)	11 (10.6%)	14 (15.4%)	0 (0%)	0.126
Cognitive symptoms	71 (32.9%)	43 (41.3%)	24 (26.4%)	4 (19.0%)	0.031
Clinical dementia diagnosis	36 (16.7%)	23 (22.1%)	13 (14.3%)	0 (0%)	0.033
Current or previous regular smoking	35 (16.2%)	19 (18.3%)	10 (11.0%)	6 (28.6%)	0.105
Lobar ICH	107 (49.5%)	58 (55.8%)	49 (53.8%)	0 (0%)	<0.001
Deep ICH	36 (16.7%)	0 (0%)	17 (18.7%)	19 (90.5%)	<0.001
Cerebellar ICH	2 (0.9%)	0 (0%)	0 (0%)	2 (9.5%)	0.003
Cortical subarachnoid hemorrhage	42 (19.4%)	28 (26.9%)	14 (15.4%)	0 (0%)	0.008
cSS present	123 (56.9%)	78 (75.0%)	45 (49.5%)	0 (0%)	<0.001
cSS classification					<0.001
Focal	35 (16.2%)	16 (15.4%)	19 (20.9%)	0 (0%)	
Disseminated	88 (40.7%)	64 (61.5%)	24 (26.4%)	0 (0%)	
Transient focal neurological episode	51 (23.6%)	34 (32.7%)	17 (18.7%)	0 (0%)	0.002
Periventricular WMH					<0.001
Fazekas 0	18 (8.3%)	7 (6.7%)	5 (5.5%)	6 (28.6%)	
Fazekas 1	69 (31.9%)	43 (41.3%)	20 (22.0%)	6 (28.6%)	
Fazekas 2	70 (32.4%)	33 (31.7%)	30 (33.0%)	7 (33.3%)	
Fazekas 3	59 (27.3%)	21 (20.2%)	36 (39.6%)	2 (9.5%)	
Deep WMH					<0.001
Fazekas 0	21 (9.7%)	8 (7.7%)	5 (5.5%)	8 (38.1%)	
Fazekas 1	74 (34.3%)	42 (40.4%)	27 (29.7%)	5 (23.8%)	
Fazekas 2	67 (31.0%)	34 (32.7%)	27 (29.7%)	6 (28.6%)	
Fazekas 3	54 (25.0%)	20 (19.2%)	32 (35.2%)	2 (9.5%)	
Deep lacune	43 (19.9%)	0 (0%)	35 (38.5%)	8 (38.1%)	<0.001
Basal ganglia PVS					<0.001
0	42 (19.4%)	29 (13.4%)	9 (4.2%)	4 (1.85%)	
1	83 (38.4%)	50 (23.1%)	24 (11.1%)	9 (4.2%)	
2	57 (26.4%)	24 (11.1%)	31 (14.4%)	2 (0.9%)	
3	19 (8.8%)	0 (0%)	16 (7.4%)	3 (1.4%)	
4	11 (5.1%)	0 (0%)	10 (4.6%)	1 (0.5%)	
N/A	4 (1.85%)				
Centrum semiovale PVS					<0.001
0	20 (9.3%)	5 (2.3%)	7 (3.2%)	8 (3.7%)	
1	33 (15.3%)	14 (6.5%)	14 (6.5%)	5 (2.3%)	
2	37 (17.1%)	16 (7.4%)	15 (6.9%)	6 (2.8%)	
3	70 (32.4%)	34 (15.7%)	36 (16.7)	0 (0%)	
4	52 (24.1%)	34 (15.7%)	18 (8.3%)	0 (0%)	
Not available	4 (1.85%)				
Lobar CMB					<0.001
0	31 (14.4%)	8 (7.7%)	9 (9.9%)	14 (66.7%)	
1–4	40 (18.5%)	17 (16.3%)	16 (17.6%)	7 (33.3%)	
≥5	145 (67.1%)	79 (76.0%)	66 (72.5%)	0 (0%)	
Deep CMBs					<0.001
0	140 (64.8%)	104 (100%)	28 (30.8%)	8 (38.1%)	
1–4	50 (23.1%)	0 (0%)	43 (47.3%)	7 (33.3%)	
≥5	26 (12.0%)	0 (0%)	20 (22.0%)	6 (28.6%)	
Left ventricular mass (American Society Echocardiography), g	161.3 (54.1)	148.8 (44.9)	168.7 (55.3)	190.8 (72.9)	<0.001

CAA indicates cerebral amyloid angiopathy; CMB, cerebral microbleed; cSS, cortical superficial siderosis; ICH, intracerebral hemorrhage; PVS, perivascular space; cSVD, cerebral small vessel disease; and WMH, white matter hyperintensity.

Clinical and neuroimaging characteristics of the patients included are shown in Table 1.

### Comparison of LVM According to MRI Classification

**Figure 3:**
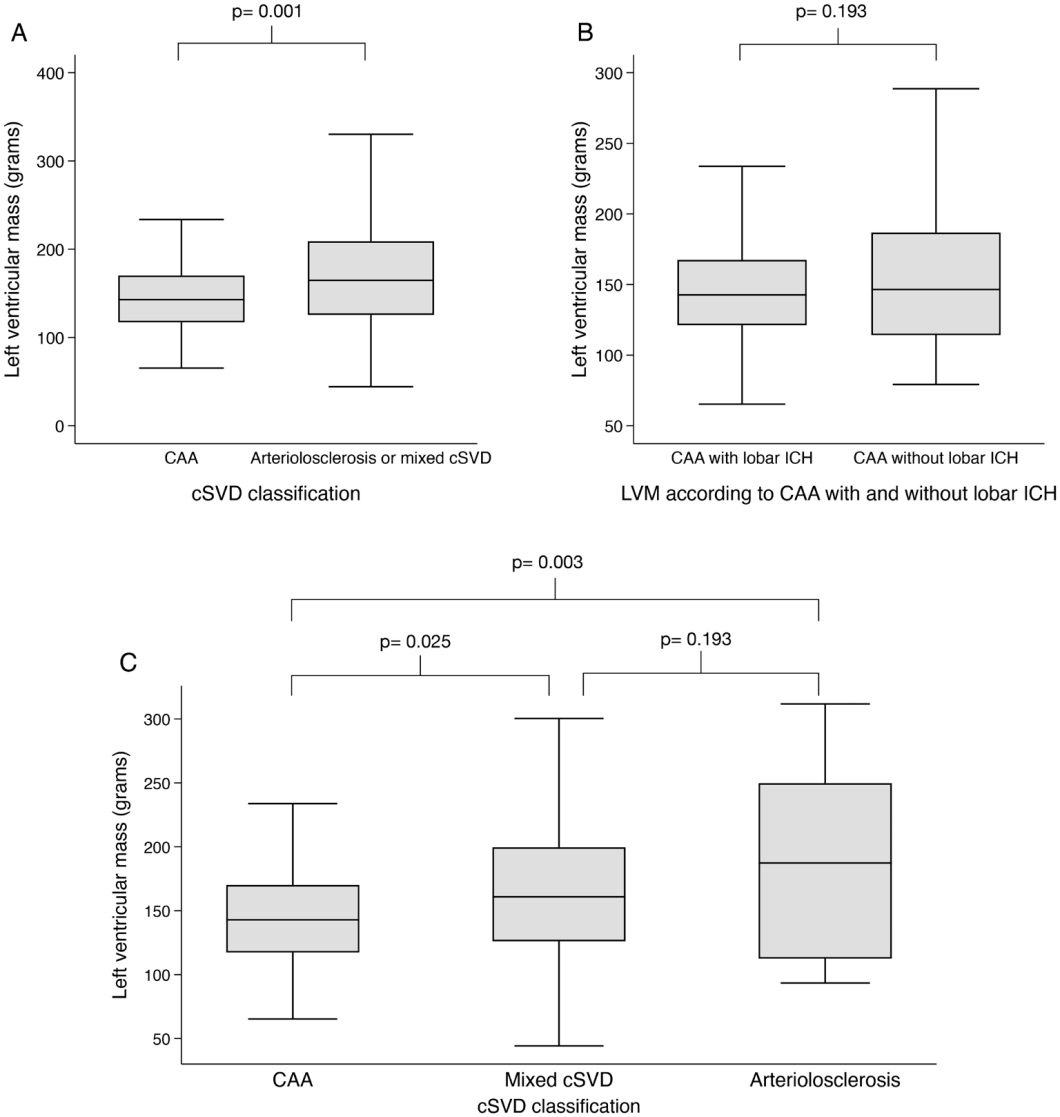
Comparison of LVM according to SVD classification. **A**, LVM according to cSVD classification (CAA vs arteriolosclerosis or mixed cSVD). **B**, LVM according to CAA with or without lobar ICH. **C**, LVM according to SVD classification (CAA vs mixed pattern cSVD v arteriolosclerosis). CAA indicates cerebral amyloid angiopathy; cSVD, cerebral small vessel disease; ICH, intracerebral hemorrhage; and LVM, left ventricular mass.

Patients with CAA only (n=104) had a significantly lower mean LVM (148.8±44.9 g) compared with patients with mixed cSVD or arteriolosclerosis (n=112) (172.8±59.3 g) (*P*=0.001) (Figure 3A).

In the subgroup of patients with CAA (n=104), patients with macroscopic ICH (n=58) showed no significant difference in mean LVM (143.7±36.7 g) compared with patients without macroscopic ICH (n=46) (155.3±53.1 g) (*P*=0.193) (Figure 3B).

LVM showed a graded increase across cSVD phenotypes from CAA (148.8±44.9 g) to mixed pattern cSVD (168.7±55.3 g) to arteriolosclerosis (190.8±72.9 g) (Figure 3C). In comparison to the CAA group, the mean LVM was significantly higher in both the mixed pattern cSVD group (*P*=0.025) and arteriolosclerosis group (*P*=0.003).

In univariable linear regression analysis (Table 2), sSVD type (with CAA as the reference category) was positively associated with LVM (for mixed cSVD β =19.87 [95% CI, 4.99–34.75]; for arteriolosclerosis β=42.02 [95% CI, 17.21–66.82; and for mixed cSVD or arteriolosclerosis β=24.02, 95% CI, 9.84–38.21]). LVM was also associated with hypertension (β=29.38 [95% CI, 15.09–43.66], *P*≤0.001) and coronary/peripheral artery disease (β=17.87 [95% CI, 0.68–35.06]). Age (β=−1.26 [95% CI, −1.91 to −0.60]) and female sex (β=−54.72 [95% CI, −67.85 to −41.60]) were inversely associated with LVM.

**Table 2:** Regression Coefficients From Univariable Models of Associations With LVM

	β coefficient	95% CI	*P* value
Age	−1.26	−1.91 to −0.60	<0.001
Sex, female	−54.72	−67.85 to −41.60	<0.001
Hypertension	29.38	15.09 to 43.66	<0.001
Diabetes	−5.57	−31.22 to 20.08	0.669
Dyslipidemia	−8.67	−24.77 to 7.44	0.290
Coronary/peripheral artery disease	17.87	0.68 to 35.06	0.042
Current/previous alcohol excess	13.20	−5.25 to 31.65	0.160
Current/previous smoking	2.17	−17.56 to 21.89	0.829
SVD category			0.001
CAA	Ref		
Mixed pattern cSVD	19.87	4.99 to 34.75	
Arteriolosclerosis	42.02	17.21 to 66.82	
SVD category			0.001
CAA	Ref		
Mixed cSVD or arteriolosclerosis	24.02	9.84 to 38.21	

CAA indicates cerebral amyloid angiopathy; cSVD, cerebral small vessel disease; and LVM, left ventricular mass.

We constructed 2 multiple linear regression models for cSVD classification: in Model 1 we compared mixed pattern cSVD and arteriolosclerosis to CAA, and in Model 2 we compared mixed pattern cSVD or arteriolosclerosis to CAA. Both models were adjusted for age, sex, and hypertension. In Model 1 (Table 3), compared with CAA, the β coefficients were 15.14 (95% CI, 1.97–28.21) and 10.54 (95% CI, −13.37 to 34.46) for mixed pattern cSVD and arteriolosclerosis respectively, *P* for trend=0.079. In model 2, compared with CAA, mixed pattern cSVD or arteriolosclerosis was independently associated with LVM (β=14.57 [95% CI, 1.74–27.4], *P*<0.026, Table 4), indicating a greater LVM for the group with mixed pattern cSVD or arteriolosclerosis than the group with CAA, adjusted mean difference +14.6 (1.7–27.4) g.

**Table 3:** Regression Coefficients From Multivariable Model 1

	β coefficient	95% CI	*P* value
Age	−0.95	−1.56 to −0.34	0.024
Sex, female	−51.47	−64.08 to −38.87	<0.001
Hypertension	15.24	2.21 28.26	0.022
cSVD category			0.079
Cerebral amyloid angiopathy	Reference		
Mixed pattern cerebral SVD	15.14	1.97 to 28.21	
Arteriolosclerosis	10.54	−13.37 to 34.46	

CAA indicates cerebral amyloid angiopathy; and cSVD, cerebral small vessel disease.

**Table 4:** Regression Coefficients From Multivariable Model 2

	β coefficient	95% CI	*P* value
Age	−0.91	−1.48 to −0.33	0.002
Sex, female	−51.22	−63.74 to −37.70	<0.001
Hypertension	14.98	20.5 to 27.92	0.023
cSVD category			0.026
Cerebral amyloid angiopathy	Reference		
Mixed cerebral SVD or arteriolosclerosis	14.57	1.74 to 27.40	

Patient characteristics with a univariable *P* value of <0.1 included with stepwise backward elimination until all variables in the model met a *P* value <0.1.

cSVD indicates cerebral small vessel disease.

Tables S1 and S2 show the association between LVM and cSVD classification in ordinal and binary logistic regression models respectively. There was a significant association between LVM per 10 g and cSVD classification, after adjusting for age, sex, and hypertension. LVM per 10 g predicted a shift from CAA to mixed pattern cSVD or a shift from mixed pattern cSVD to pure arteriolosclerosis with an odds ratio (OR) of 1.065 (95% CI, 1.002–1.132). Similarly, LVM per 10 g predicted that the underlying cSVD was mixed pattern cSVD or arteriolosclerosis rather than CAA with an OR of 1.081 (95% CI, 1.009–1.157).

## DISCUSSION

Our main finding was that patients with CAA had a significantly lower mean LVM compared with patients with mixed pattern cSVD or arteriolosclerosis, including in multivariable analyses adjusted for potential confounding factors such as hypertension. These findings suggest that cardiac structure (possibly mediated by the burden of previous exposure to systemic hypertension) is related to the phenotype of cerebral cSVD, with potential implications for ICH diagnosis, classification, understanding of pathophysiology and prevention. Our findings imply a gradient of increasing LVM as the cSVD phenotype moves from CAA to mixed pattern cSVD and then arteriolosclerosis, supporting the concept of a continuous spectrum of hemorrhagic cSVD with a differential relationship to systemic hypertension and cardiac structure.

The mechanistic implication is that exposure to systemic hypertension is an increasingly important risk factor as the cSVD phenotype shifts from CAA to arteriolosclerosis (ie, from superficial leptomeningeal and cortical arterioles to deep perforating arterioles). Our observations are consistent with previous studies that have also shown that LV enlargement is more common in non‐CAA ICH than in CAA‐ICH.^22^, ^23^

LV structure could thus potentially be a biomarker of the type of dominant cSVD pathology in patients with ICH, particularly where brain MRI is unavailable. LVH is a general predictor of mortality and is related to hypertension, though normal blood pressure does not exclude it (LVH was identified in ~3% of normotensive (≤140/90 mm Hg) individuals in 1 study [n=209]).^24^ Further studies are needed to clarify the diagnostic and prognostic relevance of abnormally high LVM or LVH in patients with ICH.

The lack of a significant difference in LVM between patients with CAA with and without macroscopic ICH suggests that in CAA, macroscopic ICH might not be related to the burden of long‐term systemic hypertensive end‐organ damage, and additional secondary ICH‐specific prevention measures in CAA need to be developed. Nevertheless, blood pressure control remains important in CAA, as demonstrated by previous studies showing that blood pressure lowering may reduce the risk of recurrent ICH in probable CAA.^25^, ^26^ Furthermore, antihypertensive treatment may be beneficial for patients with CAA, not only to reduce ICH recurrence but also to prevent the higher mortality and morbidity associated with LVH‐related cardiovascular disease. Angiotensin‐converting enzyme inhibitors may be the most effective medication for LVH regression.^27^

Our study has limitations. The small number of participants with arteriolosclerosis (n=21) could mean that our estimate of LVM is imprecise in this subgroup, possibly limiting the interpretation of this analysis. Body weight and height were not consistently recorded for all participants, so we could not normalize LVM to body surface area. We analyzed consecutive patients referred to a specialist clinic, which may have introduced selection bias because those with disabling ICH may have been excluded. Furthermore, we did not try to validate LVM as a diagnostic test for the underlying arteriopathy because we did not have access to gold‐standard neuropathology data. The results of this study are therefore exploratory and require confirmation in subsequent studies.

Strengths of our study include the detailed phenotyping of cSVD on imaging through using well‐established rating scales to ensure reliability and reproducibility. Further, we used standardized measures of LVM based on published cutoff values.

### Conclusions

In summary, we found evidence to suggest that arteriolosclerosis and mixed pattern cSVD pathology, compared with CAA, are associated with a higher LVM in patients with symptomatic hemorrhagic cSVD. The underlying mechanisms are likely to be related to greater hypertension‐related small vessel injury to higher‐pressure vessels perfusing deep regions of the brain compared with the lower pressure superficial leptomeningeal and cortical vessels. Our findings suggest that LVM might usefully be incorporated into future classifications of ICH cause.

## Sources of Funding

None.

## Disclosures

David J. Werring has received: grant funding from the Stroke Association and British Heart Foundation; speaking honoraria from Bayer; speaking and chairing honoraria from AstraZeneca and NovoNordisk; and consultancy fees from Bayer and Alnylam. Rhys P.D. Inward is supported by the Oxford‐NaturalMotion Graduate Scholarship from the University of Oxford and the European Union Horizon 2020 project MOOD (874850). The remaining authors have no disclosures to report.

## Supporting information

Tables S1 and S2

STROBE Checklist
