# Supplementary material for: Cardiac Structure Relates to Hemorrhagic Cerebral Small Vessel Disease Phenotype
Source: J Am Heart Assoc. 2026 Feb 11;15(4):e039474. doi: 10.1161/JAHA.124.039474 (PMC13055780; doi:10.1161/JAHA.124.039474)
Supplement: Supplementary file 1 — Tables S1 and S2 [file JAH3-15-e039474-s001.pdf]

SUPPLEMENTAL MATERIAL

Table S1. Ordinal logistic regression analysis for CAA v mixed pattern SVD v arteriolosclerosis according to patient characteristics and LVM

**Predictor**	**Odds ratio**	**95% CI**	**p-value**
Age group*			<0.001
18-50	Ref.		
51-60	0.383	0.096, 1.525	
61-70	0.157	0.045, 0.540	
71-80	0.086	0.026, 0.286	
>80	0.104	0.027, 0.409	
Sex	1.337	0.695, 2.574	0.385
Hypertension	3.442	1.870, 6.335	<0.001
Left ventricular mass per 10 g	1.065	1.002, 1.132	0.044

*Age group replaced age to satisfy the proportional odds assumption

Table S2. Binary logistic regression analysis for CAA versus mixed pattern cSVD or arteriolosclerosis according to patient characteristics and LVM

**Predictor**	**Odds Ratio**	**95% CI**	**p-value**
Age	0.963	0.934, 0.992	0.013
Sex, Female	1.849	0.916, 3.734	0.087
Hypertension	3.179	1.733, 5.832	<0.001
Left ventricular mass per 10 g	1.081	1.009, 1.157	0.027
